# From Diabetes to Dermatology: The Role of Teneligliptin in Bullous Pemphigoid Development

**DOI:** 10.2174/0115733998370106250610103519

**Published:** 2025-10-14

**Authors:** Md. Sadique Hussain, Gurusha Bahl, Yumna Khan, Ajay Singh Bisht, Gaurav Gupta

**Affiliations:** 1 Uttaranchal Institute of Pharmaceutical Sciences, Uttaranchal University, Dehradun, Uttarakhand, 248007, India;; 2 Department of Medical Oncology, Mahatma Gandhi Medical College and Hospital, Jaipur, 302022, Rajasthan, India;; 3 Institute of Biotechnology and Genetic Engineering (Health Division), The University of Agriculture, Peshawar, 25000, Khyber Pakhtunkhwa, Pakistan;; 4 School of Pharmaceutical Sciences, Shri Guru Ram Rai University, Patel Nagar, Dehradun, 248001, Uttarakhand, India;; 5 Centre for Research Impact & Outcome, Chitkara College of Pharmacy, Chitkara University, Rajpura, Punjab, 140401, India;; 6 Centre of Medical and Bio-allied Health Sciences Research, Ajman University, Ajman, UAE

**Keywords:** Bullous pemphigoid, teneligliptin, DPP-4 inhibitors, autoimmunity, type 2 diabetes, hemidesmosomal proteins

## Abstract

Bullous pemphigoid (BP) is an autoimmune blistering disease that especially presents in elderly patients, but recently, its incidence has increased in patients treated with inhibitors of dipeptidyl peptidase-4 (DPP-4), among which teneligliptin is widely prescribed as an antidiabetic drug for the treatment of type 2 diabetes mellitus (T2DM). Recent studies describe the association of teneligliptin with the onset of BP, hypothesizing that DPP-4 inhibition could induce immune dysregulation able to trigger autoimmune responses against skin antigens BP180 and BP230. This mini-review discusses the immunomodulatory effects of teneligliptin, immune responses, and chronic inflammation associated with diabetes that predispose patients to BP. It also discusses the clinical implications, such as monitoring for BP in T2DM patients on teneligliptin therapy. Elucidation of this relationship may help in optimizing management strategies by highlighting discontinuation or alternative therapies in the case of a high risk of development of BP.

## 1 INTRODUCTION

Bullous pemphigoid (BP) is a chronic autoimmune blistering skin disease predominantly affecting the elderly and is characterized by subepidermal blistering resulting from autoantibodies directed against the hemidesmosomal proteins BP180 and BP230 of the skin and mucous membranes [1, 2]. Over the past 20 years, numerous cases of BP have been observed, with its incidence increasing by 1.9 to 4.3 times [3]. Recognized triggers for BP include ultraviolet exposure, mechanical trauma, contrast material injections, thermal burns, insect bites, radiotherapy, surgical procedures, and a history of psoriasis [4]. Although the exact cause of BP is not known, several factors have been identified as inducing agents that initiate the disease process, including medications, infections, and other systemic conditions [1, 5, 6]. Notably, an increasing number of reports have suggested that dipeptidyl peptidase-4 inhibitors (DPP4i), one of the common pharmacological approaches to type 2 diabetes mellitus (T2DM) management, may be associated with the development of BP [7].

Teneligliptin is one of the commonly used DPP4i, which has recently been implicated in the onset of BP [8]. Although it manages T2DM effectively and is widely used, several studies and pharmacovigilance reports have pointed out the potential association of teneligliptin with bullous pemphigoid, thereby raising concern among clinicians [9]. Although the exact mechanisms of teneligliptin-induced BP are not fully known, hypotheses suggest modulation of immune responses where a drug can alter the balance of the immune system, hence inducing autoimmune responses in susceptible individuals [10].

Diabetes and autoimmune diseases are connected in a very complex manner, with chronic inflammation and immune dysregulation caused by diabetes serving as potential triggers for diseases, such as BP [11, 12]. Since T2DM is becoming increasingly prevalent worldwide and the prescription of DPP4i is very common, it is necessary to know the exact mechanisms involved in teneligliptin-related BP [13]. The early identification of teneligliptin-induced BP may lead to better management strategies and improved patient outcomes by timely discontinuing or switching drugs [14, 15]. This review critically discusses the available evidence regarding the association of teneligliptin with changes in the BP profile, considering plausible pathophysiological mechanisms and clinical implications for management.

## 2 METHODOLOGY

In order to ensure a comprehensive and unbiased synthesis of the existing literature regarding the association between teneligliptin and bullous pemphigoid (BP), we employed a structured search strategy across multiple databases. A systematic literature search was conducted on PubMed, Scopus, Embase, and Web of Science databases up to November 2024. The search terms used included combinations of keywords, such as “teneligliptin”, “dipeptidyl peptidase-4 inhibitors”, “DPP-4 inhibitors”, “bullous pemphigoid”, “autoimmune blistering diseases”, and “adverse drug reactions”. Both medical subject headings (MeSH) and free-text terms were utilized to maximize retrieval sensitivity. The inclusion criteria encompassed peer-reviewed original research articles, review articles, case reports, case series, observational studies, meta-analyses, and pharmacovigilance analyses specifically focusing on teneligliptin-induced BP or DPP-4 inhibitor-induced BP where the data on teneligliptin were available. Letters, correspondence data, non-English publications, and studies lacking relevance to teneligliptin or BP were excluded. Additionally, we reviewed reports from pharmacovigilance databases, such as the Japanese Adverse Drug Event Report (JADER) and the FDA Adverse Event Reporting System (FAERS), for signal detection analysis related to teneligliptin. Any discrepancies in study inclusion were resolved by discussion among the authors. This systematic approach allowed us to present a focused and reliable narrative, highlighting the current evidence, clinical implications, and future research regarding teneligliptin's potential role in BP development.

## 3 THE LINK BETWEEN DPP-4 INHIBITORS AND BP

Recent literature suggests that DPP4i, like teneligliptin, may be associated with the development of BP (Table **1**) [9, 16, 17]. One of the studies has reported that the non-FDA-approved DPP4i has a significantly higher reporting odds ratio (ROR) of inducing BP than FDA-approved DPP4i. This is probably a consequence of the relative underreporting of the adverse effects of the non-FDA-approved drugs in the FDA Adverse Event Reporting System (FAERS) [16]. Furthermore, one more study confirmed the association between DPP4i and increased risk of the development of BP. However, it should be noted that the overall possibility of this adverse effect remains low. In clinical practice, DPP4i continues to be recognized as safe and effective for the management of T2DM [16, 17].

While the precise mechanism underlying the association between teneligliptin and BP remains incompletely understood, emerging evidence suggests that immune dysregulation and the elicitation of autoimmune responses may play a critical role. One proposed mechanism is that DPP4is contribute to the accumulation of incretin hormones, such as glucagon-like peptide-1 (GLP-1), having immunomodulatory effects. This altered hormonal balance may influence immune cell activity, leading to disruptions in T-cell regulation, increased autoreactive B-cell activation, and the subsequent production of autoantibodies targeting the basement membrane zone, which is a hallmark of BP pathogenesis [18].

Although direct mechanistic evidence specifically linking teneligliptin to BP development is limited, recent studies provide indirect support for this association. A meta-analysis of randomized controlled trials (RCTs) suggested the potential role of DPP4is, including teneligliptin, in increasing BP risk [19]. Additionally, observational studies indicate an elevated incidence of pemphigoid among DPP4i users, further reinforcing the need for mechanistic investigations to establish causality. The role of teneligliptin in modifying immune responses, particularly through effects on cytokine signaling, T-helper cell differentiation, and complement system activation, warrants further study using *in vitro* models and patient-derived immune profiling.

Given these findings, a cautious approach is warranted for individuals undergoing teneligliptin therapy, particularly those with predisposing factors for autoimmune diseases. Clinicians should remain vigilant for early dermatological symptoms indicative of BP, and timely dermatologic referral is crucial for prompt diagnosis and intervention. Future research should focus on large-scale epidemiological studies, mechanistic immunological analyses, and proteomic investigations to clarify the precise role of teneligliptin in BP pathogenesis [20].

People with BP currently on teneligliptin treatment have many other options that could be used, and there are several other medicines that one could adopt as an alternative. However, whether to continue the same medication or shift to other medications depends on the history and present status of the patient. People suffering from BP may consult their physician extensively to receive a proper diagnosis and specific treatment advice. In the prospective study of 253 BP patients, the multi-center BLISTER trial carefully weighed the benefits and risks of initiating treatment with either doxycycline or prednisolone [21]. Several topical corticosteroids, systemic corticosteroids, immunosuppressants, and even some newer biologics are available for use in the treatment of BP. It is important to underline that any medication prescribed to influence BP requires close medical supervision to provide maximum safety and effectiveness throughout therapy.

## 4 TENELIGLIPTIN: MECHANISM OF ACTION AND THERAPEUTIC ROLE

Clinical trials have documented that teneligliptin is effective not only as monotherapy, but also in combination with metformin, glimepiride, or pioglitazone. In 12- or 16-week placebo-controlled phase 2 and 3 trials, once-daily administration of 20 mg and 40 mg doses of teneligliptin caused significant improvements in glycaemic control. It has also shown efficiency in patients with terminal renal failure, which has even further expanded its use in diabetic patients with a wide range of renal functions. Most treatment-emergent adverse events during these studies were mild in intensity and thus led to relatively few discontinuations. Long-term studies, along with extension phases, as long as 52 weeks, provided evidence for the durability of such glycaemic control changes with no emerging safety concerns. These findings represent the therapeutic potential of teneligliptin in T2DM management and its tolerability [37].

Teneligliptin was developed by Mitsubishi Tanabe Pharma and marketed in collaboration with Daiichi Sankyo in September 2012 in Japan. It is approved in several countries, including Japan, Argentina, Korea, and India. The distinctive J-shaped or anchor-locked domain structure allows teneligliptin to inhibit the DPP-4 enzyme potently [38]. This inhibition is important because DPP-4 inactivates incretin hormones responsible for the maintenance of glucose homeostasis. By inhibiting DPP-4, teneligliptin increases active incretin hormone levels, thus enhancing insulin secretion in response to meals, suppressing glucagon release, and together maintaining the balance of blood sugar [39]. Furthermore, teneligliptin has been found to effectively control glycemic parameters by showing a good safety profile and without adjusting the dosage in renal impairment conditions [40-43].

One of the major distinctive features of teneligliptin compared to other members of DPP4i is the peculiar chemical structure, which provides higher possibilities of closer and longer interaction with the enzyme DPP-4 and contributes to drug efficacy [37]. Figure (**1**) depicts a comparison of teneligliptin with other DPP4is. Moreover, it has also proven to be effective in those patients in whom other DPP4is have not been effective in providing adequate glycemic control. Thus, it has proven to be an effective alternative for those patients needing a more aggressive approach to their disease management. In various clinical trials, teneligliptin has proven to be significantly effective in reducing HbA1c levels and bringing overall glycemic improvement in patients suffering from T2DM. One prospective observational study with a duration of 52 weeks showed that in patients with the switch to teneligliptin from other DPP4is, there was a better glycemic outcome, underscoring its safety and effectiveness during an extended period [44].

Teneligliptin, like other DPP-4is, is generally well tolerated, with a low incidence of hypoglycemia and a neutral effect on body weight. However, differences in pharmacokinetic properties and molecular structures may contribute to variations in the side-effect profiles. In clinical trials, teneligliptin has been reported to demonstrate a similar safety profile to other DPP-4is, with the most common adverse effects being mild gastrointestinal symptoms (nausea, diarrhea), upper respiratory tract infections, and headaches [45]. Notably, unlike saxagliptin, which has been associated with a higher risk of heart failure hospitalizations in some studies [46], teneligliptin has not demonstrated an increased cardiovascular risk in long-term follow-ups [47].

When comparing dermatological adverse effects, BP has been increasingly reported in association with DPP-4is, though the frequency varies among different agents. Several pharmacovigilance studies have suggested that vildagliptin and teneligliptin have a higher reported incidence of BP than sitagliptin and linagliptin [48]. The structural differences among DPP-4is may influence their immunogenic potential, with teneligliptin’s extended half-life and high DPP-4 selectivity possibly contributing to altered immune responses leading to autoantibody production. A meta-analysis of DPP-4i-associated BP cases found vildagliptin to have the highest risk, followed by teneligliptin, while linagliptin exhibited the lowest BP risk [49].

In addition to BP, infection risk varies among DPP-4is. Some studies indicate that saxagliptin and sitagliptin may slightly increase the risk of urinary tract and respiratory infections due to their immunomodulatory effects, whereas teneligliptin does not appear to significantly elevate infection susceptibility [50]. Similarly, while most DPP-4is are considered weight-neutral, vildagliptin and saxagliptin have been linked to marginal weight gain, whereas teneligliptin maintains a stable weight profile, making it a preferable choice for obese patients with T2DM [47]. These comparisons highlight the nuanced safety differences among DPP-4is, emphasizing the importance of individualized treatment selection. Given the higher BP risk associated with certain DPP-4is, including teneligliptin, clinicians should monitor patients closely for dermatological symptoms, particularly in elderly populations and those with predisposing autoimmune conditions. Future head-to-head comparative studies and pharmacogenetic investigations are needed to elucidate the mechanisms behind DPP-4i-associated BP and optimize safer prescribing practices.

## 5 POTENTIAL MECHANISMS OF TENELIGLIPTIN IN TRIGGERING OR EXACERBATING BULLOUS PEMPHIGOID

A range of skin-related side effects, such as bullous, exfoliative, urticarial, and exanthematous reactions, have been linked to the use of DPP4i, especially teneligliptin. DPP4 is found within the skin, yet the precise mechanisms connecting teneligliptin intake and BP remain unknown.

Several mechanisms have been described to explain the potential role of teneligliptin in triggering or exacerbating BP. One proposed explanation involves the increased activity of proinflammatory chemokines, like eotaxin, which leads to eosinophil activation in the skin and results in blister formation. Guliani *et al.* noted this as the first reported case linking teneligliptin with BP [51]. Saha *et al.* reported another case where, according to the WHO-UMC scale, teneligliptin was likely the cause of BP [3]. Another case reported by Pareek S noted that bullous pemphigoid was further aggravated by teneligliptin intake [52].

Another hypothesis suggests that teneligliptin may exert an influence on the immune system through modulation in the expression of DPP-IV on the surface of immune cells involved in the autoimmune response in patients with active BP [51, 53]. Other mechanisms include the production of autoantibodies against the BP antigens, especially BP180 and BP230, which are constituents of hemidesmosomes in the skin. Increased detection of anti-BP180 NC16A autoantibodies has been recorded following the use of teneligliptin, which provides a suggestion that the drug might promote the autoimmune reaction against these skin components [53, 54].

Furthermore, the chronic inflammatory state itself induced by diabetes mellitus may predispose the patients to autoimmune diseases. The introduction of teneligliptin to diabetic patients may further create such an environment, which could trigger BP in susceptible individuals [51, 54]. Although teneligliptin is effective in maintaining the blood glucose level within a physiological range, its potential to develop or worsen BP demands caution while monitoring, with further investigation required to understand its underlying mechanism and provide necessary risk mitigation measures associated with its use.

The exact mechanisms are not fully understood, but the inhibition of DPP-4 affects various immune cells and pathways. DPP-4 is expressed on the surface of several immune cells, including T cells. Its inhibition can alter the balance between cytokines and the response to immune stimuli [55]. This disturbance in immune regulation can potentially lead to autoimmune reactions against the skin and, consequently, to conditions, such as BP [19]. Meta-analyses of RCTs have confirmed this association, pointing out an increased incidence of BP among patients treated with DPP4is [19]. Further, case reports and pharmacovigilance studies have strengthened this association between these drugs and the induction of autoimmune blistering diseases [56]. Continuous observation and studies of such adverse effects are required to increase knowledge regarding the extended impact of DPP4is on the immune system and develop strategies that could mitigate such risks.

Clinical trials have established that teneligliptin generally has a good safety profile with a low risk of hypoglycemia and is weight-neutral, and hence is a preferred choice as an add-on therapy for glycemic control [57]. Compared to other DPP4is, teneligliptin has been reported to possess a similar safety profile, with only a few adverse effects observed throughout prolonged administration [58]. Notably, it has demonstrated a low tendency for QT/QTc prolongation, an adverse effect generally associated with most anti-diabetic drugs [57]. Kishimoto's comparative study indicated that teneligliptin and sitagliptin, another DPP4i, share consistent safety and efficacy, although the longer half-life of teneligliptin might offer an advantage in glucose fluctuations [45, 58]. However, given the potential for variability in patient responses, continuous monitoring for adverse effects remains essential. While teneligliptin is generally well-tolerated, emerging reports linking DPP-4 inhibitors to autoimmune blistering diseases, including BP, underscore the need for large-scale epidemiological studies and meta-analyses to assess its long-term dermatological safety (Table **2**). Expanding pharmacovigilance efforts and conducting RCTs will be critical in determining whether teneligliptin poses a distinct risk for BP development compared to other DPP-4is.

## 6 DEMOGRAPHIC FACTORS INFLUENCING TENELIGLIPTIN-ASSOCIATED BULLOUS PEMPHIGOID RISK

While BP is predominantly observed in elderly populations, emerging evidence suggests that various demographic factors, including age, ethnicity, comorbidities, and sex, may influence the risk and clinical presentation of BP in patients receiving teneligliptin. BP primarily affects individuals over the age of 70, likely due to age-related immune senescence, which increases the propensity for autoantibody formation and chronic inflammation. The use of teneligliptin in this age group may further modulate immune responses, exacerbating the risk of BP development. However, isolated cases of BP have also been reported in younger diabetic patients on DPP-4is, suggesting that additional factors, such as genetic susceptibility, metabolic dysregulation, and pre-existing autoimmune conditions, may contribute to disease onset [64, 65]. Large-scale epidemiological studies are needed to determine whether teneligliptin accelerates BP onset in younger individuals or whether its effects are more pronounced in elderly populations.

Ethnicity appears to play a role in BP susceptibility, with studies suggesting that Asian populations may have a higher incidence of DPP-4i-associated BP compared to Caucasian populations. A pharmacovigilance study using the Japanese Adverse Drug Event Report (JADER) database indicated a significant association between DPP-4 inhibitors and BP, particularly in elderly Japanese patients [66]. One potential explanation for these regional differences is genetic predisposition, particularly in HLA polymorphisms. The HLA-DQB1*03:01 allele has been strongly associated with BP risk in both European and East Asian populations, raising the possibility that certain genetic backgrounds may predispose individuals to teneligliptin-induced immune dysregulation [5]. Future pharmacogenomic studies are warranted to confirm these associations and explore whether genetic screening can help identify high-risk patients before initiating teneligliptin therapy.

Comorbid conditions, particularly autoimmune diseases, may further modulate BP risk in patients using teneligliptin. Several studies have suggested that individuals with autoimmune disorders, such as rheumatoid arthritis, systemic lupus erythematosus, and thyroid disease, may be at heightened risk of BP due to pre-existing immune dysregulation (Amber *et al.*, 2018). Additionally, metabolic comorbidities frequently seen in type 2 diabetes patients, including obesity, chronic kidney disease, and cardiovascular disease, may further contribute to immune dysfunction, thereby increasing the likelihood of BP development. As DPP-4 inhibitors influence inflammatory pathways, these conditions could amplify the risk of an aberrant autoimmune response (Tasanen *et al.*, 2019) [55]. Given these findings, clinicians should be particularly cautious when prescribing teneligliptin to patients with multiple comorbidities and ensure regular dermatological monitoring for early detection of BP symptoms.

While BP has traditionally been reported to have a male predominance, some studies suggest that drug-induced BP, including cases associated with DPP-4 inhibitors, may exhibit a more balanced gender distribution (Plaquevent *et al.*, 2019) [63]. This raises the possibility that hormonal and immunological differences between sexes could modulate the risk of BP development in teneligliptin users, though further research is required to clarify these potential associations. Overall, recognizing the demographic variability in BP risk among teneligliptin users is crucial for developing targeted prevention strategies.

Future large-scale cohort studies should systematically evaluate whether specific ethnic groups, age demographics, or patients with underlying comorbidities exhibit an increased risk of teneligliptin-associated BP. Additionally, pharmacogenetic screening for susceptibility markers, such as HLA-DQB1*03:01, could pave the way for personalized diabetes management by identifying individuals at heightened autoimmune risk before initiating DPP-4i therapy [67]. Until more definitive data are available, healthcare providers should adopt a proactive monitoring strategy, incorporating regular dermatological assessments every 3-6 months for patients on teneligliptin, especially those with predisposing autoimmune conditions. Patients should be educated about the early signs of BP, including pruritus, urticarial lesions, and blister formation, and instructed to seek immediate medical evaluation if symptoms develop. If BP is suspected, prompt diagnostic confirmation *via* direct immunofluorescence, BP180/BP230 ELISA, and histopathological analysis is essential.

In cases where BP is confirmed, discontinuation of teneligliptin should be the first step, followed by transitioning to alternative anti-diabetic therapies, such as SGLT2 inhibitors, GLP-1 receptor agonists, or metformin-based regimens. For BP management, topical corticosteroids remain the mainstay for mild cases, whereas systemic corticosteroids, doxycycline, or immunosuppressive agents (*e.g.*, methotrexate, azathioprine, rituximab) may be required for moderate-to-severe presentations. Long-term follow-up with antibody titer monitoring and periodic dermatologic evaluation is recommended to assess disease remission and prevent relapse. By implementing personalized risk assessment, vigilant monitoring, and evidence-based treatment adjustments, clinicians can mitigate the risk of teneligliptin-associated BP while ensuring optimal glycemic control for patients with type 2 diabetes. Future research should focus on clarifying the mechanistic underpinnings of this drug-induced autoimmune response, identifying additional genetic risk factors, and developing predictive models for safer patient stratification.

## 7 LIMITATIONS AND FUTURE DIRECTIONS

Despite increasing reports linking DPP-4is, including teneligliptin, to BP, the current understanding of this association remains incomplete due to several limitations in available studies. Most published data are derived from case reports, retrospective cohort analyses, and pharmacovigilance databases, which inherently introduce reporting bias and confounding variables [66]. The absence of large-scale, prospective clinical trials restricts the ability to establish a definitive causal relationship between teneligliptin and BP. Additionally, significant heterogeneity exists across studies regarding sample sizes, patient demographics, diagnostic criteria, and follow-up durations, making it difficult to generate consistent risk estimates [49]. Another major limitation is the unclear pathophysiological mechanisms underlying teneligliptin-associated BP. While it has been proposed that DPP-4 inhibition may modulate T-cell activation, complement system regulation, and cytokine balance, direct experimental evidence linking teneligliptin to BP180/BP230 autoantibody formation remains lacking [64]. Additionally, pharmacogenetic factors, such as HLA-DQB1*03:01 polymorphisms, which have been associated with an increased risk of BP in certain ethnic populations, have not been systematically studied in teneligliptin users [67]. This genetic variability might partly explain the higher prevalence of DPP-4i-associated BP in Asian populations compared to Western cohorts [66].

From a clinical perspective, the absence of standardized screening protocols for early BP detection in teneligliptin users presents a significant challenge. Given the increasing reports of DPP-4i-associated BP, routine dermatological monitoring in high-risk individuals, particularly elderly patients or those with predisposing autoimmune conditions, should be considered. The lack of comparative safety analyses among different DPP-4is further limits the ability to determine whether teneligliptin poses a higher BP risk than other drugs in its class [66]. Addressing these knowledge gaps requires well-designed prospective, multicenter cohort studies to quantify the relative risk of BP across different DPP-4is while controlling for genetic predisposition and comorbidities. Additionally, mechanistic research using *in vitro* and *in vivo* models is essential to clarify how DPP-4 inhibition influences immune pathways leading to BP development. Finally, pharmacogenetic investigations focusing on HLA-associated risk markers could pave the way for a personalized approach to diabetes management, enabling clinicians to identify patients at higher risk before initiating teneligliptin therapy.

Future research should prioritize large-scale epidemiological studies, mechanistic investigations, and pharmacogenomic analyses to provide more definitive insights and inform safer prescribing practices for patients with T2DM. Until more robust data emerge, clinicians should exercise vigilance when prescribing teneligliptin, particularly in high-risk populations, and promptly evaluate any dermatologic symptoms suggestive of BP.

## CONCLUSION

Teneligliptin, used as one of the effective drugs in the management of glycemic levels among T2DM patients, has also been studied for its possible association with BP, an autoimmune skin condition characterized by a response against skin-specific antigens. While the exact mechanism has not yet been established, hypotheses have pointed out that DPP4i can cause an imbalance in immune homeostasis and enhance autoimmunity, leading to the development of BP. Patients on teneligliptin, especially elderly individuals with chronic inflammation due to diabetes, may face an elevated BP risk, necessitating vigilant clinical monitoring for early signs of blistering and prompt intervention if BP develops. Discontinuing teneligliptin and considering alternative treatments may mitigate risk and improve patient outcomes. As BP incidence continues to rise among DPP4i users, further research is essential to elucidate the precise immunopathological mechanisms linking teneligliptin to autoimmune blistering diseases. Future studies should focus on *in vitro* and *in vivo* investigations of DPP-4 inhibition in immune regulation, large-scale epidemiological analyses to assess patient susceptibility, and pharmacogenetic research to identify genetic risk factors. Additionally, clinical trials and case-control studies are needed to refine guidelines for safer, individualized T2DM management, particularly for patients at higher risk of BP development.

## Figures and Tables

**Fig. (1):**
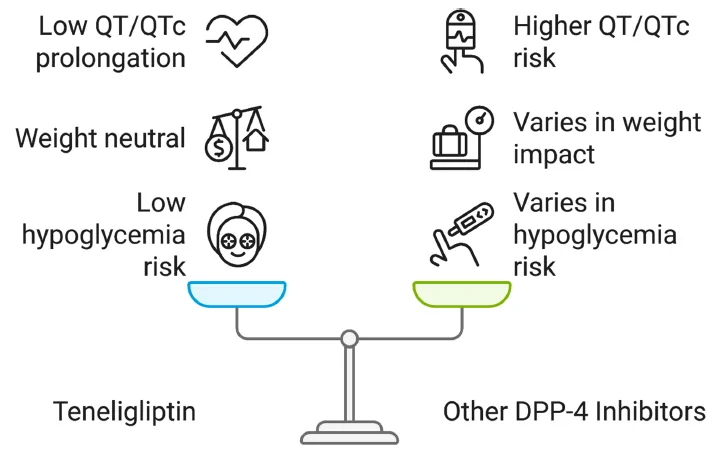
Evaluation of teneligliptin and other DPP-IV inhibitors.

**Table 1:** Year-wise selected case reports of DD-4 inhibitors-associated bullous pemphigoid development.

**Year**	**Cases**	**DPP-4 Inhibitor Used (n)**	**Latency Time (Months)**	**References**
**2011**	2	Vildagliptin (2)	2	[22]
**2012**	6	Vildagliptin (5), sitagliptin (1)	2-13	[23]
**2013**	3	Vildagliptin (1), sitagliptin (2)	5-6	[24]
**2014**	1	Sitagliptin (1)	12	[25]
**2015**	3	Vildagliptin (3)	1-37	[26]
**2016**	3	Vildagliptin (2), linagliptin (1)	2-3	[14]
**2016**	1	Vildagliptin (1)	12	[27]
**2016**	2	Linagliptin (2)	3-4	[28]
**2017**	1	Linagliptin (1)	9	[29]
**2017**	1	Linagliptin (1)	5	[30]
**2017**	1	Sitagliptin (1)	3	[31]
**2017**	1	Anagliptin (1)	1	[32]
**2017**	9	Vildagliptin (4), sitagliptin (5)	5-48	[33]
**2018**	5	Vildagliptin (1), linagliptin (2), Sitagliptin (1), anagliptin (1)	1-15	[34]
**2018**	5	Vildagliptin (1), sitagliptin (1), Linagliptin (2), alogliptin (1)	1-8	[35]
**2019**	10	Vildagliptin (4), sitagliptin (5), linagliptin (1)	5-24	[36]

**Table 2:** Epidemiological studies of gliptin-associated bullous pemphigoid.

**Country**	**Cases/Controls (n)**	**Mean Age (Cases), y**	**Adjusted OR (95% CI)**	**References**
Switzerland	23 (DM+BP)/170 (DM)	77.6	DPP-4i: 2.48 (0.75-8.3)	[33]
France	61 (BP+DM)/122 (DM)	79.1	DPP-4i: 2.64 (1.19-5.85)	[59]
			Vildagliptin: 3.57 (1.07-11.84)	
			Sitagliptin: 2.13 (0.77-5.89)	
			Linagliptin/saxagliptin: 2.90 (0.47-17.74)	
Finland	3,397/12,941	76.6	DPP-4i: 2.19 (1.55-3.11)	[60]
			Vildagliptin: 10.4 (4.56-23.80)	
			Sitagliptin: 1.37 (0.93-2.01)	
			Metformin: 1.05 (0.88-1.24)	
Japan	168 cases (DPP4i-BP: 32, non-DPP4i-BP: 136)	79.7	NA	[61]
Israel	82 (BP+DM)/328 (DM)	79.1	DPP-4i: 3.16 (1.86-5.37)	[62]
			Vildagliptin: 10.67 (5.09-22.36)	
			Linagliptin: 6.65 (2.24-19.72)	
			Sitagliptin: 0.42 (0.12-1.45)	
France	1,787 / 225,412	77.9	NA	[63]
Korea	670 (BP+DM)/670 (DM)	75.3	DPP-4i: 1.58 (1.25-2.00)	[17]
			Vildagliptin: 1.81 (1.31-2.50)	
			Sitagliptin: 1.70 (1.19-2.43)	
			Linagliptin: 1.64 (1.15-2.33)	
			Other DPP-4is: 1.25 (0.73-2.14)	

**Abbreviations: ** BP: Bullous pemphigoid; CI: Confidence interval; DM: Diabetes mellitus; DPP-4i: Dipeptidyl peptidase-4 inhibitor; NA: Not available; OR: Odds ratio.

## LIST OF ABBREVIATIONS

BP: Bullous Pemphigoid
T2DM: Type 2 Diabetes Mellitus
MeSH: Medical Subject Headings
JADER: Japanese Adverse Drug Event Report
RCTs: Randomized Controlled Trials
ROR: Reporting Odds Ratio
